# Ceramide sphingolipid signaling mediates Tumor Necrosis Factor (TNF)-dependent toxicity via caspase signaling in dopaminergic neurons

**DOI:** 10.1186/1750-1326-7-45

**Published:** 2012-09-13

**Authors:** Terina N Martinez, Xi Chen, Sibali Bandyopadhyay, Alfred H Merrill, Malú G Tansey

**Affiliations:** 1 Department of Physiology, The University of Texas Southwestern Medical Center at Dallas, 6001 Forest Park Rd., Dallas, TX, 75390, USA; 2 Department of Physiology, Emory University School of Medicine, 615 Michael St., Atlanta, GA, 30322, USA; 3 School of Biology and the Parker H. Petit Institute for Bioengineering and Bioscience, Georgia Institute of Technology, 315 Ferst Drive, Atlanta, GA, 30332-0363, USA

**Keywords:** Neuroinflammation, TNF, Ceramide, Sphingolipids, Neuronal apoptosis, Neurodegeneration, ER stress, Caspase, Akt

## Abstract

**Background:**

Dopaminergic (DA) neurons in the ventral midbrain selectively degenerate in Parkinson’s disease (PD) in part because their oxidative environment in the substantia nigra (SN) may render them vulnerable to neuroinflammatory stimuli. Chronic inhibition of soluble Tumor Necrosis Factor (TNF) with dominant-negative TNF inhibitors protects DA neurons in rat models of parkinsonism, yet the molecular mechanisms and pathway(s) that mediate TNF toxicity remain(s) to be clearly identified. Here we investigated the contribution of ceramide sphingolipid signaling in TNF-dependent toxicity.

**Results:**

Ceramide dose-dependently reduced the viability of DA neuroblastoma cells and primary DA neurons and pharmacological inhibition of sphingomyelinases (SMases) with three different inhibitors during TNF treatment afforded significant neuroprotection by attenuating increased endoplasmic reticulum (ER) stress, loss of mitochondrial membrane potential, caspase-3 activation and decreases in Akt phosphorylation. Using lipidomics mass spectrometry we confirmed that TNF treatment not only promotes generation of ceramide, but also leads to accumulation of several atypical deoxy-sphingoid bases (DSBs). Exposure of DA neuroblastoma cells to atypical DSBs in the micromolar range reduced cell viability and inhibited neurite outgrowth and branching in primary DA neurons, suggesting that TNF-induced *de novo* synthesis of atypical DSBs may be a secondary mechanism involved in mediating its neurotoxicity in DA neurons.

**Conclusions:**

We conclude that TNF/TNFR1-dependent activation of SMases generates ceramide and sphingolipid species that promote degeneration and caspase-dependent cell death of DA neurons. Ceramide and atypical DSBs may represent novel drug targets for development of neuroprotective strategies that can delay or attenuate the progressive loss of nigral DA neurons in patients with PD.

## Background

The exact molecular mechanisms that contribute to pathogenesis in Parkinson’s disease (PD) have not been well delineated; many different cellular processes have been implicated in PD, including diminished function of the ubiquitin proteasome system, generation of reactive oxygen species, endoplasmic reticulum (ER) stress, compromised mitochondrial function and protein aggregation (Reviewed by [1]). Additionally, inflammation and activated microglia have been generally implicated in PD pathology [2-7] and increased levels of pro-inflammatory cytokines such as TNF, IL-1β and IL-6, have been observed in the cerebral spinal fluid (CSF) and striatum of PD patients relative to healthy age-matched controls [8]. Furthermore, gene polymorphisms in inflammatory genes (TNF-308 and IL-1β-511) have been associated with an increased risk of developing PD [9]. Specifically, we have previously reported that blocking soluble TNF (solTNF) signaling with novel dominant-negative TNF inhibitors attenuates loss of dopaminergic neurons both *in vitro* and *in vivo*[10]. Soluble TNF signals through the canonical transmembrane death receptor TNF receptor 1 (TNFR1) to potently transduce inflammatory stimuli [11,12]. TNFR1 is constitutively expressed by most cell types, including DA neurons, which are acutely sensitive to TNF-induced toxicity [13-15]. However, TNFR1 can elicit signaling through numerous down-stream effectors, including p38, JNK, MAPK, and ceramide (Reviewed by [16]) but identification of specific pathways required for TNF-induced cytotoxicity in DA neurons has not yet been forthcoming.

The aim of this study was to test the hypothesis that ceramide signaling cascades are an important effector arm of TNF-mediated cytotoxicity in DA neurons. Ceramide is a sphingolipid with a well-established role in cell membrane homeostasis [17]. However, a wealth of studies over the past decade established the role of ceramide and its downstream metabolites as second messenger sphingolipids due to their rapid and transient generation in cells and their ability to modulate a variety of physiologic and stress responses [18-20]. Specifically, ceramide has been implicated in the cell death pathway activated by the death domain receptor ligands TNF and Fas-L [21,22]. Additionally, ceramide has been shown to activate apoptosis in primary cortical neurons [23] and in primary neuronal cultures from embryonic mesencephalon [24], but its role as a critical downstream effector of TNF-induced apoptosis in DA neurons has not been fully delineated. To explore the role of ceramide signaling in the TNF-dependent cytotoxicity of DA neurons, we used both primary neuronal cultures from embryonic rat ventral mesencephalon and the MN9D dopamine neuron-like cell line [25] which is a hybridoma line derived from fusion of murine embryonic ventral mesencephalon and neuroblastoma cells and is often used as an *in vitro* model of DA neurons [26,27] because the cells express high levels of tyrosine hydroxylase (TH), the rate limiting enzyme in dopamine biosynthesis, and efficiently synthesize, store and release dopamine [28]; additionally, their sensitivity to oxidative stress and inflammatory stimuli is similar to that of primary DA neurons from ventral midbrain [25-27]. Here we report that TNF and ceramide exert dose-dependent cytotoxic effects on DA neuroblastoma cells and primary DA neurons. Functionally, inhibitors of SMase activity which block sphingomyelin hydrolysis and ceramide generation attenuated TNF-induced cytotoxicity, decreases in phospho-Akt, increases in caspase 3 cleavage as well as mitochondrial membrane potential changes, and ER stress in DA cells. Ultimately, the mechanisms of TNF-induced cytotoxicity in DA cells culminated in and were found to be completely dependent on caspase signaling, suggesting a model in which ceramide/sphingolipid signaling cascades are key effectors of TNF-dependent apoptotic death in DA neurons. Our data also revealed that TNF treatment not only activates sphingomyelinases (SMases) to produce ceramide but also leads to generation of several other atypical deoxy-sphingoid bases (DSBs) including desoxymethylsphingosine (1-desoxyMeSo), deoxysphinganine (deoxySa), and desoxymethylsphinganine (1-desoxyMeSa); when added exogenously *in vitro*, some of these DSBs inhibit neurite outgrowth and are toxic to DA neurons*.* These findings suggest that multiple sphingolipid mediators may be responsible for mediating TNF neurotoxicity and identification of specific sphingolipid metabolites may reveal opportunities for drug development to delay or prevent DA neuron degeneration.

### Experimental procedures

#### Primary and Cell Line Cultures

The MN9D dopaminergic neuroblastoma cell line was developed by Dr. Alfred Heller [25] and was a generous gift from Dr. Michael Zigmond, at the University of Pittsburgh. MN9D cells were grown in culture in sterile complete media (CM) which consisted of: high glucose (4,500 mg/L) Dulbecco’s Modified Eagle Medium (DMEM, Sigma, D5648) dissolved in sterile tissue culture tested water (Sigma) supplemented with 10% fetal bovine serum (FBS, Hyclone Fetal Clone III), sodium bicarbonate (3.7 g/L, Sigma), 25 mM HEPES (Sigma), and 1% Penicillin/Streptomycin (Sigma) at a final pH of 7.3 in a humidified 5% CO_2_ incubator at 37°C. MN9D cell cultures were seeded in 75 cm^2^ tissue culture flasks (Costar) and plated at a density of 7,500 cells per well for 96-well plates (100 μL CM per well); 35,000 cells per well (500 μL CM per well) for 24-well plates; and 50,000 cells per well (2 mL CM per well) for 6-well plates. After plating and allowing attachment of cells overnight in CM, MN9D cells were differentiated for 72 hrs via a complete media change to differentiation media (DM) which contained serum free DMEM (same CM as above, except FBS was excluded) supplemented with 5 mM 2-Propylpentanoic acid (valproic acid, Sigma, P6273) and 1X N2 supplement (Invitrogen) as described in previous protocols [26].

Primary mixed neuron-glia cultures from rat ventral mesencephalon (rat MES) were prepared as described previously [10]. Briefly, ventral mesencephalic tissues were dissected from embryonic day 14 (E14) Fischer 344 rats and dissociated with mild mechanical trituration. Cells were plated into 96-well culture plates pre-coated with poly-D-lysine (0.1 mg/mL) and laminin (20 g/mL) at a density of 2.5 x10^5^ cells/mL in DMEM/F12 supplemented with 10% FBS, 1 g/L glucose, 2 mM L-glutamine, 1 mM sodium pyruvate, 100 M non-essential amino acids, 50 U/mL penicillin, 50 g/mL streptomycin, and 10 ng/mL basic fibroblast growth factor (bFGF). Cultures were maintained at 37°C in a humidified atmosphere of 5% CO_2_/95% air. Cultures were replenished 2 days later with 100 μL/well fresh medium lacking bFGF and were treated 5 days after plating. For treatment, the cultures were maintained in 100μL/well of medium supplemented with 2.5% FBS and lacking bFGF.

#### MTS Metabolic Assays

Treated diff-MN9D cells in 96-well plates were evaluated for overall viability using the MTS assay (Promega, CellTiter 96 AQueous One Solution Cell Proliferation Assay) according to the manufacturer’s instructions. Twenty microliters (20 μL) of the MTS reagent was added to cell cultures with DM-containing treatments and/or inhibitors. The cells were incubated with the MTS reagent at 37°C, 5% CO_2_ for 2 hrs prior to colorimetric quantification of MTS reduction into a blue formazan by-product by metabolically active cells. The absorbance of blue formazan was measured at 492 nm wavelength using a Multiskan Ascent absorbance plate reader (Thermo Labsystems).

#### LDH Release Cytotoxicity Assay

Treated diff-MN9D cells in 96-well plates were evaluated for cytotoxicity using an LDH release assay (Clontech Laboratories, Mountain View, CA) as per the manufacturer’s instructions. LDH reactions were measured at a wavelength of 492 nm on an absorbance plate reader (Thermo Lab Systems Multiskan Ascent). The maximum LDH activity was determined by lysing the cells with 1% Triton X-100.

#### Neurite Length and Branching Studies

After treatment with the specified sphingolipids, primary rat mesencephalic (MES) cultures were fixed with 4% paraformaldehyde, stained with anti-MAP2 (1:1000, Millipore, MAB3418) and anti-TH antibodies (1:1000, Millipore, AB152) and counterstained by DAPI. Images were captured on an IMAGEXPRESS 5000A automated cellular imaging and analysis system. When analyzing the morphology of TH-positive neurons, the neurite outgrowth application module of MetaXpress software was used and multi-parameter analysis measurements were performed.

#### Sphingomyelinase and Ceramide Synthesis Inhibition

Diff-MN9D cells in 96-well plates were pre-treated in triplicate or quadruplicate with one of four different pharmacological inhibitors: the acid sphingomyelinase (ASMase) inhibitor desipramine HCl, used at 1 μM and 5 μM, (Sigma, D3900, dissolved in sterile H_2_O), the neutral sphingomyelinase (NSMase) inhibitor GW4869, used at 10 μM, and 20 μM, (Calbiochem, No. 567715, dissolved in DMSO, aliquotted and stored under argon gas), the synthetic bisphosphonate sSMase inhibitor 7c, also known as ARC39, used at 1 μM, (a generous gift from Dr. Christoph Arenz, Humboldt University in Berlin, Germany), the serine palmitoyltransferase inhibitor myriocin (a generous gift from Dr. Philip Scherer, UT Southwestern Medical Center at Dallas), dissolved in ethanol and used at 10 μM or the ceramide synthase inhibitor Fumonisin B1, used at 50 μM (Axorra LLC, No.: 350-017-M001, dissolved in sterile H_2_O). Diff-MN9D cells were pre-treated with ceramide inhibitors or control diluents for 30 minutes via a 50% media change with DM that contained a 2X concentration of the respective inhibitor or control diluent (i.e. 50 μL of the initial 100 μL DM was removed and replaced with 50 μL of DM containing a 2X concentration of a ceramide inhibitor for a final volume of 100 μL with 1X inhibitor). After pre-treatment with ceramide inhibitors for 30 minutes, TNF was added by a 1:100 dilution of a TNF stock concentration into media that contained ceramide inhibitors (1 μL of 100X TNF was added to 100 μl DM). In the case of GW4869, which declines in effective NSMase-inhibition over time [29] the GW4869 reagent was added to DM 30 minutes prior to TNF treatment by a 50% media change and was then re-added 24 hrs after initial GW4869 pre-treatment by addition of a 1:100 dilution of a GW4869 stock concentration (1 μL of 100X GW + 100 μL DM to equal 1X) into DM already containing TNF treatments. Diff-MN9D cells were incubated at 37°C, 5% CO_2_ for 48 hrs post TNF treatment prior to determination of cell viability by MTS assay.

#### Western blots for ER stress, caspase-3, and p-AKT activation

MN9D cells were plated on 6-well plates at the density of 50,000 cells/well. Twenty-four to forty-eight hours later, the complete media was changed into differentiation media and the cells were neurally differentiated for 72 hours. Before TNF treatment, diff-MN9D cells were pre-incubated with desipramine or GW4869 for 1 hour. After 24- hours treatment with ceramide, TNF, TNF/Des or GW, cell lysates were collected in 200 uL SDS-PAGE loading buffer. When running SDS-PAGE, 15ul of sample lysate was loaded in each well. GAPDH and α-Tubulin were used as controls for densitometry quantification. The quantified data shown represent at least three independent experiments.

#### Cytofluorometric Analysis of Mitochondrial Membrane Potential

Mitochondrial membrane potential in diff-MN9D cells was measured as previously described [30]. Briefly, MN9D cells were seeded into black-walled, clear-bottomed 24-well plates onto Poly-L-Lysine (PLL) coated (Sigma, P2636, MW = 30,000-70,000, 1 mg/mL) Assistent glass cover slips (12 mm, No.0, distributed by Carolina Biological Supply) at a density of 35,000 cells per well in 500 μL CM. The MN9D cells were incubated overnight at 37°C, 5% CO_2_ and were then differentiated via a complete media change with DM. After 72 hrs in DM, the diff-MN9D cell cultures were treated with C2-Cer or DMSO vehicle, or TNF or media vehicle via a complete media change with 1X treatment in DM. After incubation with C2-Cer for 18 hrs or TNF for 36 hrs, tetra methyl rhodamine methyl ester (TMRM) (Invitrogen, T668, re-suspended in DMSO) was loaded into treated diff-MN9D cells at 150 nM in warm assay buffer (AB, 500 μL per well) which consisted of: NaCl (80 mM), KCl (75 mM), D-glucose (25 mM) and HEPES (25 mM) diluted in sterile H_2_O and adjusted to a final pH of 7.4. To control for TMRM background cytofluorescence, carbonyl cyanide 3-chlorophenylhydrazone (CCCP, Sigma, C2759) was used. At the time of incubation with TMRM, 10 μM CCCP (re-suspended in DMSO) was co-added with TMRM in AB to parallel wells of diff-MN9D cells treated with TNF or C2-Cer. TMRM and TMRM/CCCP loaded cells were incubated for 15 minutes in a humidified incubator with 5% CO_2_ at 37°C prior to quantification of TMRM cytofluorescence by excitation at 544 nm wavelength and emission at 590 nm wavelength on a FLUOstar Omega plate reader (BMG Scientific). The TMRM signal in TMRM/CCCP conditions is considered background, and this signal was used to normalize TMRM cytofluorescence values for each respective TNF or C2-Cer condition.

#### Caspase Inhibition and BAPTA-AM Studies

Diff-MN9D cells in 96-well plates were treated in triplicate or quadruplicate with TNF or C2-Cer alone or were co-treated with one of two caspase inhibitors, 25 μM Z-VAD-FMK (Z-VAD, a pan caspase inhibitor, obtained from Promega), or 25 μM Z-IETD-FMK (Z-IETD, a caspase 8-specific inhibitor, obtained from R&D Systems). The treated diff-MN9D cells were incubated at 37°C, 5% CO_2_ with C2-Cer for 24 hrs or with TNF for 48 hrs prior to determination of overall cell viability via the MTS assay as described above. For BAPTA-AM studies, diff-MN9D cells were pre-loaded with the cell permeant intracellular Ca^2+^ chelator BAPTA-AM (BAPTA, 10 μM) 30 min prior to treatment with concentrations of C2-Cer. At the endpoint of the study, cell viability was assayed by MTS reduction.

#### Lipidomics for Quantitative Analysis of Complex Sphingolipids and Sphingoid Base

To perform quantitative analysis of complex sphingolipids and sphingoid bases in MN9D ventral mesencephalon DA neuroblastoma cells in response to TNF (10 ng/mL) treatment, we employed a lipidomics approach based on previously published protocols [31]. For internal standards, the Ceramide/Sphingoid Internal Standard Mixture II (LM-6005) from Avanti Polar Lipids (Alabaster, AL, USA) was used with 25 pmol of each of the following: Sphingosine (C17 base), Sphinganine (C17 base), Sphingosine-1-P (C17 base), Sphinganine-1-P (C17 base), Lactosyl(ß) C12 Ceramide, C12-Sphingomyelin, Glucosyl(ß) C12-Ceramide, C12-Ceramide, and C12-Ceramide-1-P (LM-6005).

#### Cell Treatments with TNF, Ceramide and Sphingoid Bases

After incubation in DM for 72 hours, diff-MN9D cells cultured in 96-well plates were treated in triplicate or quadruplicate by a 50% media change with DM that contained 2X TNF (recombinant mouse, R&D MT-410), C2-Ceramide (C2-Cer, N-acetyl-D-Sphingosine, Sigma A7191) or C2-dihydroceramide (C2-DH-Cer, Sigma, C7980) as a negative control for C2-Ceramide because it lacks the 4–5 *trans* bond in the sphingosine moiety and cannot activate downstream ceramide signaling [22,32]. The TNF was dissolved in sterile Phosphate Buffered Saline (PBS, Sigma) and C2-Cer and C2-DH-Cer were dissolved in DMSO (Sigma) and aliquotted and stored under argon gas. As a control in parallel treatments, a DMSO vehicle condition equivalent to the amount of DMSO in the highest concentration of C2-Cer/C2-DH-Cer was used. TNF, C2-Cer or C2-DH-Cer-treated diff-MN9D cells were incubated at 37°C, 5% CO_2_ for 72 or 48 hrs respectively, prior to being evaluated for overall viability using the MTS assay (described below). TNF, C2-Cer or C2-DH-Cer treatments of diff-MN9D cells in 24-well or 6-well plates were done in duplicate or triplicate by a complete media change from DM to DM containing 1X TNF, C2-Cer or C2-DH-Cer. Etanercept, an Fc-fusion protein consisting of TNFR2 and the Fc component of human immunoglobulin IgG1, was used as a positive control because it binds TNF and blocks its bioactivity [10,33].

Lipid-BSA stock solutions of the following sphingolipids from Avanti Polar Lipids were prepared as per published protocols [34,35]. 1-deoxysphinganine C18H39NO (Catalog # 860493), 1-desoxymethylsphinganine C17H37NO (Catalog # 860473); 1-deoxysphingosine C18H37NO (Catalog# 860470); 1-desoxymethylsphingosine C17H35NO (Catalog # 860477); C16 ceramide C34H67NO3 (Catalog # 860516); Sphingosine (d18:1) C18H37NO2 (Catalog # 860490) and Sphinganine (d18:0) C18H39NO2 (Catalog # 860498). Briefly, lipids were placed in Pyrex 13x100 mm borosilicate, screw-capped glass test tubes with Teflon caps and solubilized in a volume of ethanol to get a final concentration of 100 mM; sonication and warm tap water were employed to ensure homogenous resuspension. To make 1:1 (concentration) sphingoid base-BSA complex, 20 uL of a given sphingoid base (100 mM) in ethanol was quickly injected into a 1 mL volume of a BSA (2 mM) solution by using a Hamilton syringe. To ensure optimal complexing of the lipids to BSA, tubes were shaken vigorously and sonicated as needed. When treating the diff-MN9D cells, different concentrations of sphingoid base-BSA complexs were prepared in differentiation media and added to diff-MN9D cells and incubated for 24 hours at the concentrations indicated under “Results”. When treating the rat MES cultures, sphingoid base-BSA complexes were prepared in treatment media (DMEM/Ham F-12 with 1% Pen/strep, 1% Glutamine, 1% non-essential amino acids and 2.5% FBS) without bFGF.

#### Statistical Analyses

Statistical analyses were performed using GraphPad Prism5 software (GraphPad Prism, San Diego, CA). Intergroup differences among the means between the various dependent variables were analyzed using one-way ANOVA; when ANOVA indicated significant differences, it was followed by Tukey’s *post-hoc* group comparison test. Differences among group means between two independent variables were analyzed by two-way ANOVA, followed by Tukey’s *post-hoc* test when the ANOVA indicated significant differences. Values expressed are the group means ^+^/- standard error of the mean (SEM).

## Results

### TNF and ceramide induce cytotoxicity in differentiated MN9D cells and in primary DA neurons from ventral mesencephalon

**Figure 1:**
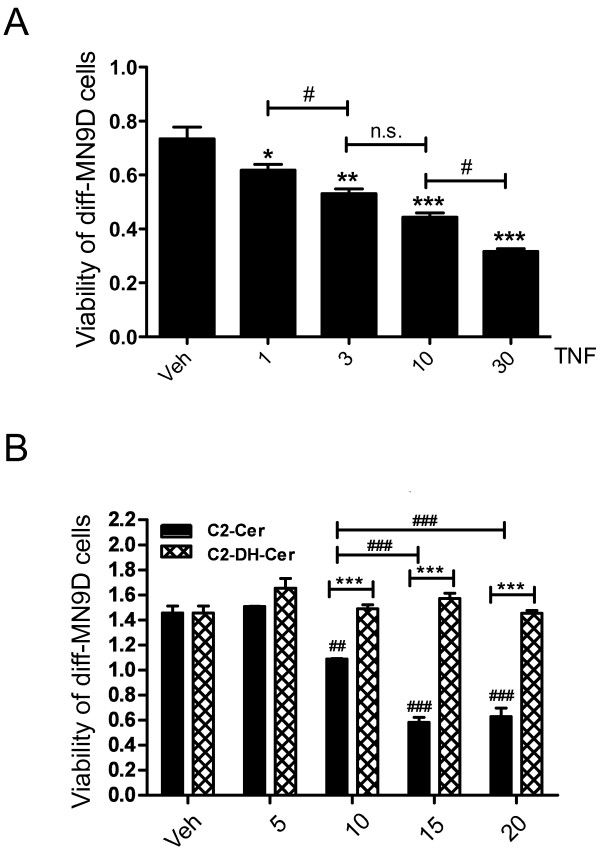
**TNF and ceramide (but not dihydro-Ceramide) reduce viability of neurally differentiated MN9D dopaminergic (DA) cells.A**, Dose-dependent cytotoxic cell death in diff-MN9D cells treated with TNF for 72 hrs. All values represent group means +/− SEM, n = 3–4. One-way ANOVA with Tukey’s post-hoc; * denotes p < 0.05, ** denotes p < 0.01, and *** denotes p < 0.001 compared to vehicle (DMSO, 1%); for comparison between doses # denotes p < 0.05, n.s. denotes not significant. **B**, Dose-dependent C2-Ceramide-induced cytotoxic death in diff-MN9D cells. Cells were treated with ceramide (C2-Cer), or with equal concentrations of C2-dihydroceramide (C2-DH-Cer) as a negative control. All values represent group means +/− SEM, n = 3–4. Two-way ANOVA with Tukey’s post-hoc test for comparing C2-Cer conditions to C2-DH-Cer conditions where **denotes p < 0.01, *** denotes p < 0.001. One-way ANOVA to test for dose-dependent cell death in C2-Cer conditions where # denotes p < 0.05, ### denotes p < 0.001 relative to vehicle or different C2-Cer conditions as indicated.

In light of our previous findings showing that ventral mesencephalon dopaminergic (DA) neurons are acutely sensitive to TNF *in vitro* and *in vivo*[10], we hypothesized that ceramide sphingolipids are critical effectors of TNF-induced cytotoxicity. First, we aimed to establish a correlation between TNF-dependent ceramide generation and the effect of TNF or ceramide exposure on the viability of neuronally differentiated MN9D cells or primary DA neurons. We found that TNF dose-dependently decreased the viability of diff-MN9D cells as measured by the MTS metabolic assay (Figure 1A). To test the hypothesis that elevated ceramide is directly toxic to diff-MN9D cells, we treated the cells with various concentrations of C2-Cer or C2-dihydroceramide (C2-DH-Cer) as a negative control; C2-DH-Cer is an analog of C2-Cer lacking the 4–5 *trans* bond in the sphingosine moiety that is incapable of activating downstream ceramide signaling [22,32]. We found that C2-Cer but not C2-DH-Cer induced dose-dependent decreases in diff-MN9D viability (Figure 1B). We previously determined that non-differentiated MN9D (non-diffMN9D) cells are not sensitive to concentrations of TNF that elicit cytotoxicity in diff-MN9D cells [26]. Similarly, C2-Cer was not cytotoxic to non-diff-MN9D cells (Additional file 1: Figure S1).

### TNF-induced neurotoxicity in DA cells and neurons is attenuated by SMase inhibitors

**Figure 2:**
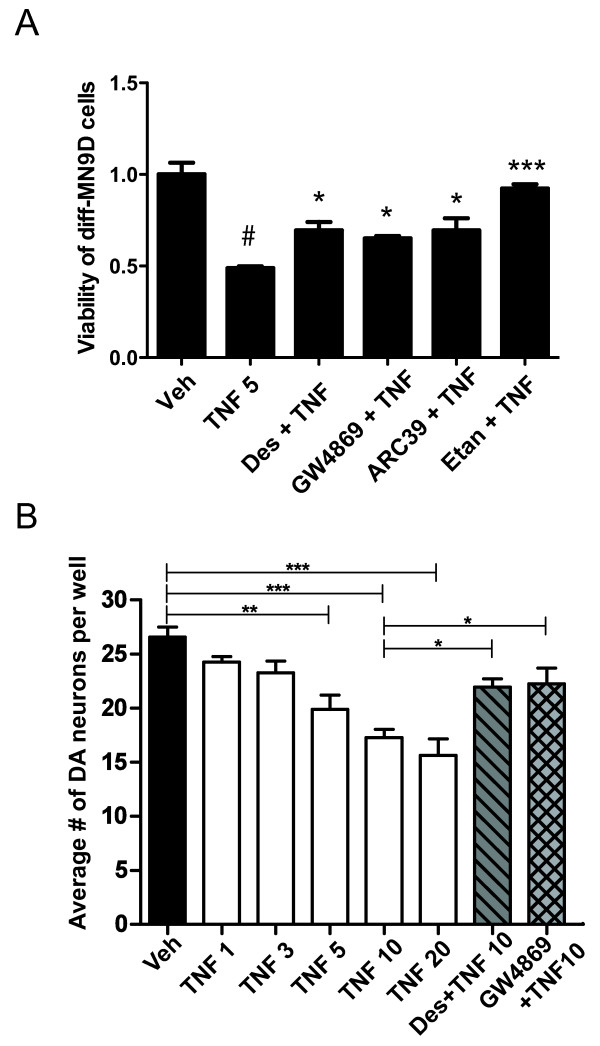
**TNF-induced neurotoxicity in DA cells and neurons is attenuated by SMase inhibitors.A**, TNF-induced cytotoxic cell death is dependent on SMase hydrolysis of sphingomyelin. Diff-MN9D cells were pre-treated for 30 minutes with 5 μM desipramine (Des) or 10 μM GW4869 or 1 μM ARC39 followed by 5 ng/mL TNF for 48 hrs prior to MTS viability assay. Cell viability was measured by the MTS assay described under Methods. All values represent group means +/− SEM, n =3 - 4. One-way ANOVA with Tukey’s post-hoc; # denotes difference between TNF and vehicle at p < 0.001, * and *** denote difference from TNF alone at p < 0.05 or p < 0.001, respectively. **B**, TNF induced dose-dependent death of primary ventral mesencephalon DA neurons with SMase inhibitors affording robust rescue; 5 μM Desipramine (Des); 10 μM GW4869. All values represent group means +/− SEM, n =3 - 4. One-way ANOVA, Tukey’s post-hoc test to compare the extent of dose-dependent cell death in response to increasing concentrations of TNF and two-way ANOVA with Tukey’s post-hoc test to compare inhibitor conditions to 10 ng/mL TNF without inhibitors. * denotes p < 0.05, ** denotes p < 0.01, *** denotes p < 0.001.

Ceramide can be generated either through a *de novo* biosynthesis pathway involving several enzymatic reactions downstream of the initial condensation of serine and palmitoyl-CoA on the cytoplasmic surface of the ER or through the sphingomyelin recycling pathway whereby acid or neutral sphingomyelinases (SMases) hydrolyze sphingomyelin (SM) to ceramide [36]. We hypothesized that activation of SMases at the plasma membrane by the activated TNFR1/TNF receptor complex is the mechanism by which TNF exposure leads to ceramide signaling and cytotoxicity in DA cells. To test this hypothesis directly, we pre-treated diff-MN9D cells with different inhibitors of SMases for 30 minutes followed by treatment with TNF for 48 hrs. We pre-treated diff-MN9D cells with three different compounds that inhibit SMases with different mechanisms of action. Pre-incubation with desipramine (Des) (an inhibitor of acid sphingomyelinase, ASMase[37]), GW4869 (an inhibitor of neutral sphingomylinase, NSMase [38]), or with 7c, also known as ARC39 (an inhibitor of lysosomal and secreted ASMase [39]) at the concentrations indicated all significantly attenuated TNF-induced cytotoxicity of diff-MN9D cells as measured by the MTS assay (Figure 2A). To confirm and extend these findings, we assayed the extent to which two of these SMase inhibitors attenuated TNF-induced death of DA neurons in primary neuron-glia cultures from rat ventral mesencephalon. Consistent with the results in MN9D cells, Des and GW4869 protected primary DA neurons from TNF-induced death (Figure 2B) to an extent comparable to that achieved in previous studies using the soluble TNF-selective inhibitor XENP345 [10]. Together these pharmacological data strongly suggest that TNF-dependent activation of SMases results in SM hydrolysis and generation of ceramide that is cytotoxic to DA neurons, compromising their viability. To confirm that the ceramide-generating pathway involved in mediating TNF-dependent cytotoxicity is due to SM hydrolysis by SMases rather than through *de novo* ceramide formation, we repeated these experiments using pharmacological inhibitors of the *de novo* ceramide biosynthesis pathway. We observed that inhibition of the enzyme serine palmitoyltransferase (the rate-limiting enzyme in *de novo* ceramide biosynthesis) by myriocin or inhibition of the enzyme ceramide synthase (which converts sphinganine to dihydroceramide) by Fumonisin B1 did not mitigate TNF-induced cytotoxicity in diff-MN9D cells (Additional file 2: Figure S2). Collectively, our data support a model in which SMase hydrolysis of SM to form ceramide is requisite for TNF-induced cytotoxicity in diff-MN9D cells and DA neurons.

### *TNF and C2-Ceramide-induced cytotoxicity involves endoplasmic reticulum stress pathways*

**Figure 3:**
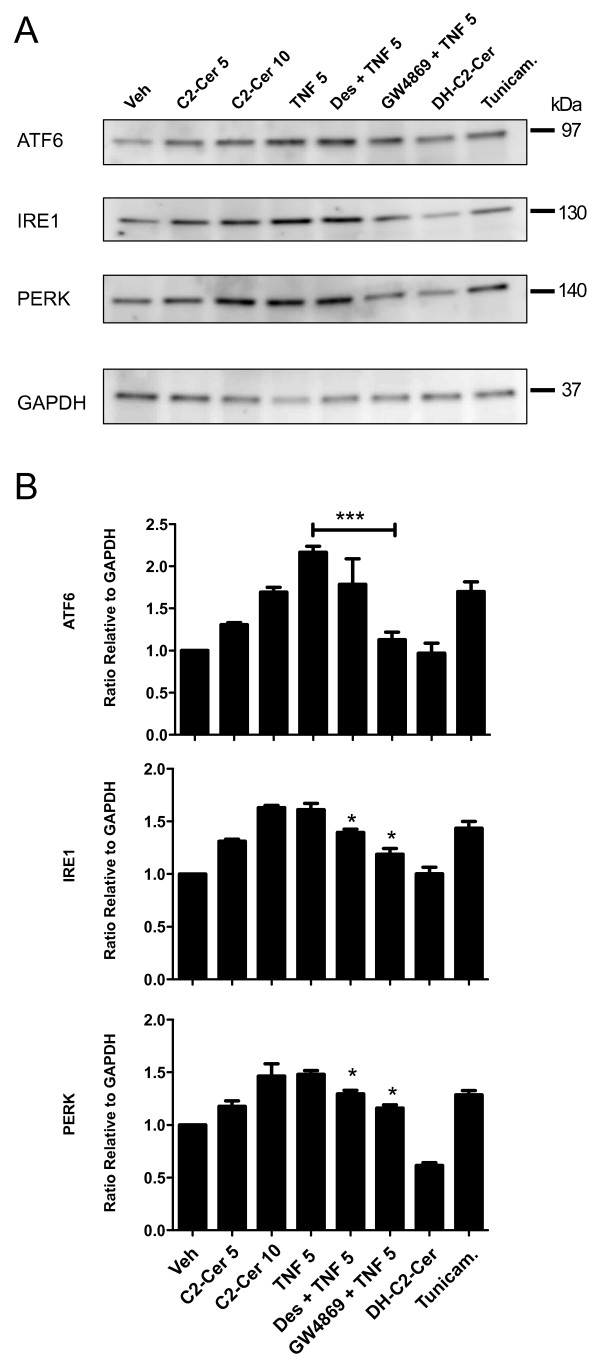
**TNF-induced ER stress responses in MN9D dopaminergic (DA) cells were attenuated by SMase inhibitors.A**, Diff-MN9D cells were pre-treated with 5 μM Desipramine (Des) or 10 μM GW4869 followed by treatment with 5 ng/mL TNF for 24 hours. Cell lysates were harvested for SDS-PAGE and were analyzed by immunoblot using antibodies against the ER stress proteins ATF6, IRE1, and PERK or GAPDH for normalization. Dihydro-ceramide (DH-C2-Cer at 10 μM) and tunicamycin (Tunicam. at 0.1 μg/mL) were used as negative and positive controls, respectively. **B**, Quantification of western blots in A. All values represent group means +/− SEM, n = 3–4. One-way ANOVA with Tukey’s post-hoc test; * denotes p < 0.05, *** denotes p < 0.001 compared to TNF alone.

TNF and ceramide have been shown to impinge on ER stress mechanisms in non-neuronal cells types [40,41] and ER stress has been implicated as a potentially important pathway in PD pathogenesis [42], being coupled to the cell death program in DA cells in response to the toxin paraquat [43]. Therefore, we investigated the extent to which activation of ER stress pathways by TNF are dependent on ceramide generation by SMase activity in diff-MN9D cells. We used immunoblots to ascertain if TNF treatment of diff-MN9D cells increased protein expression of key ER stress transducers, including activating transcription factor 6 (ATF6), ER- resident PKR-like eIF2α kinase (PERK), and inositol requiring enzyme-1 (IRE). We found that the increased expression of ER stress proteins by TNF and C2-Cer was comparable to increased protein levels caused by the positive control tunicamycin, (Figure 3) which is known to potently induce ER stress by inhibiting protein N-glycosylation [44]. These results support a model in which TNF employs ceramide signaling to elicit ER stress in DA cells.

### *TNF-induced decrease in mitochondrial membrane potential and LDH release in DA cells is ameliorated by SMase inhibitors*

**Figure 4:**
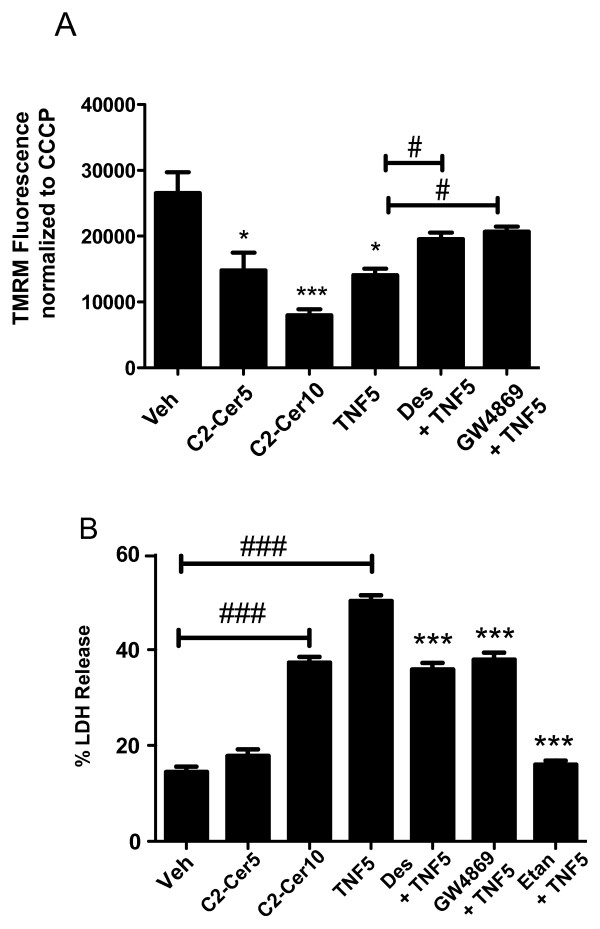
**TNF- induced decreases in mitochondrial membrane potential were attenuated by SMase inhibitors.A**, Treatment of diff-MN9D cells for 18 hrs with 5 or 10 μM C2-Cer or 5 ng/mL TNF comprised mitochondrial membrane potential. TMRM cytofluorescence was normalized to CCCP + Veh or CCCP + TNF, as CCCP uncouples mitochondrial membranes. Pre-treatment with the SMase inhibitors Desipramine or GW4869 attenuated the TNF-induced decreases in mitochondrial membrane potential. All values represent group means +/− SEM, n = 3–4. One-way ANOVA with Tukey’s post test; * denotes p < 0.05, *** denotes p < 0.001 compared to ‘Veh’; # denotes p < 0.05 compared to TNF alone. **B**, Treatment of diff-MN9D cells for 18 hrs with 5 or 10 μM C2-Cer or 5 ng/mL TNF induced cytotoxicity as measured by LDH release. Pre-treatment with SMase inhibitors (Desipramine or GW4869) attenuated TNF-induced cytotoxicity; the TNF inhibitor (etanercept) was used as a positive control. All values represent group means +/− SEM, n = 3–4. One-way ANOVA with Tukey’s post test; ### denotes p < 0.001 compared to vehicle; *** denotes p < 0.001 compared to TNF alone.

TNF has been reported to cause rapid decreases in mitochondrial membrane potential and coincident increases in reactive oxygen species [45]. Consistent with our hypothesis that ceramide is an important downstream effector of TNF cytotoxicity, ceramide itself has been shown to directly affect the mitochondrial electron transport chain [46]. To further elucidate the mechanisms of TNF and C2-Cer-induced cytotoxicity and to determine if TNF/ceramide signaling in diff-MN9D cells impinges on mitochondria, we investigated whether TNF or C2-Cer adversely impact mitochondrial membrane potential by evaluating tetramethyl rhodamine methyl ester (TMRM) cytofluorescence. TMRM is a cationic mitochondrial-selective probe that accumulates in the negatively charged mitochondrial membrane in proportion to mitochondrial membrane potential. Diff-MN9D cells treated with 5 ng/mL TNF for 36 hrs or 5 or 10 μM C2-Cer for 18 hrs exhibited compromised mitochondrial membrane potential as evidence by reduced TMRM cytofluorescence relative to vehicle treated diff-MN9D cells (Figure 4A), lending support to the interpretation that both TNF and C2-Cer adversely affect mitochondrial integrity in diff-MN9D cells. Moreover, the SMase inhibitors desipramine and GW4869 partially restored the TMRM signal in diff-MN9D cells (Figure 4A). To confirm and extend these findings we performed an additional assay to measure TNF-induced cytotoxicity. Diff-MN9D cells were treated for 18 hrs with 5 or 10 μM C2-Cer or 5 ng/mL TNF; lactate dehydrogenate (LDH) release was then measured. In agreement with results from MTS assays, pre-treatment with SMase inhibitors (Desipramine or GW4869) attenuated TNF-induced LDH release. The TNF inhibitor etanercept was used as a positive control. These data support a model in which TNF-induced cytotoxicity is mediated via ceramide-dependent signaling leading to disruption of mitochondrial membrane potential in DA cells.

### *Inhibition of SMases during TNF exposure attenuates caspase 3 cleavage in DA cells*

**Figure 5:**
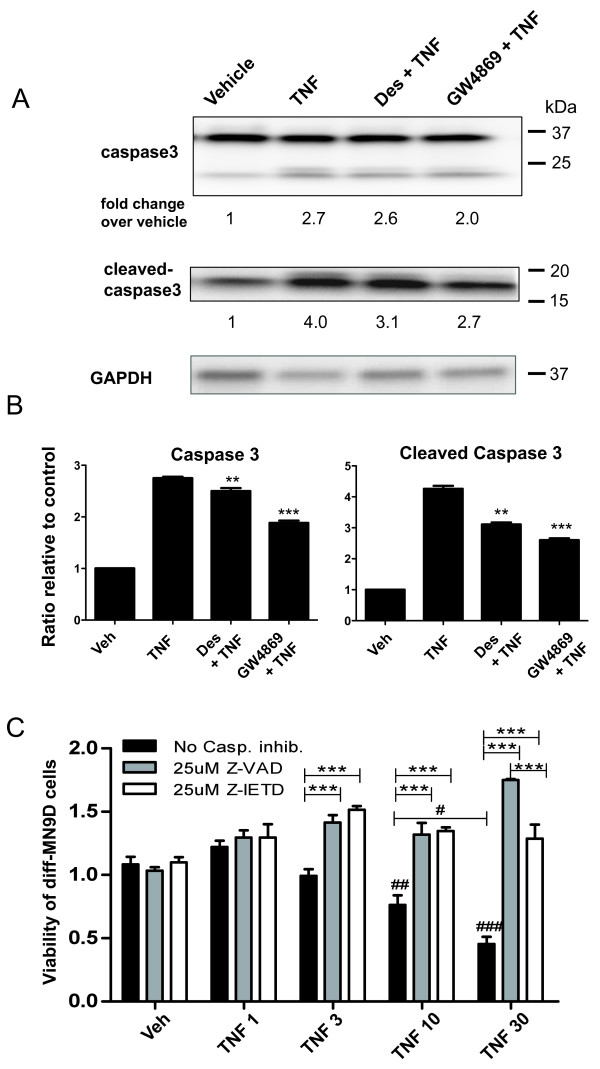
**TNF- and ceramide-induced caspase-3 cleavage was attenuated by SMase inhibitors and caspase inhibitors rescued differentiated MN9D cells from TNF-induced cytotoxicity.A**, Diff-MN9D cells were treated for 3 days with 5 ng/mL TNF in the presence or absence of the ASMase inhibitor Desipramine (Des, 5 μM) or the NSMase inhibitor GW4869 (10 μM) and were thereafter harvested for SDS-PAGE and immunoblot analysis of total caspase 3 or cleaved caspase 3. **B**, Quantification of western blot analysis of caspase 3 and cleaved caspase 3. One-Way ANOVA with Tukey’s post-hoc test to compare inhibitor conditions to TNF alone, where ** denotes p < 0.01, ***denotes p < 0.001. **C**, MTS assay for cell viability in diff-MN9D cells. TNF induced dose-dependent death of diff-MN9D cells and was caspase-dependent. Co-treatment with TNF plus the pan-caspase inhibitor Z-VAD (25 μM) or with TNF plus the caspase-8-specific inhibitor Z-IETD (25 μM) robustly blocked TNF-induced cell death in diff-MN9D cells. All values represent group means +/− SEM, n = 3–4. One-way ANOVA with Tukey’s post-hoc test to compare the effect of specified TNF concentrations on diff-MN9D viability without caspase inhibitors in the MTS assay, where # denotes p < 0.05, ## denotes p < 0.01, and ### denotes p < 0.001 compared to ‘Veh’ or between two concentrations. Two-way ANOVA with Tukey’s post-hoc to compare effects of caspase inhibitors at each TNF concentration where *** denotes p < 0.001 compared to no caspase inhibitor.

**Figure 6:**
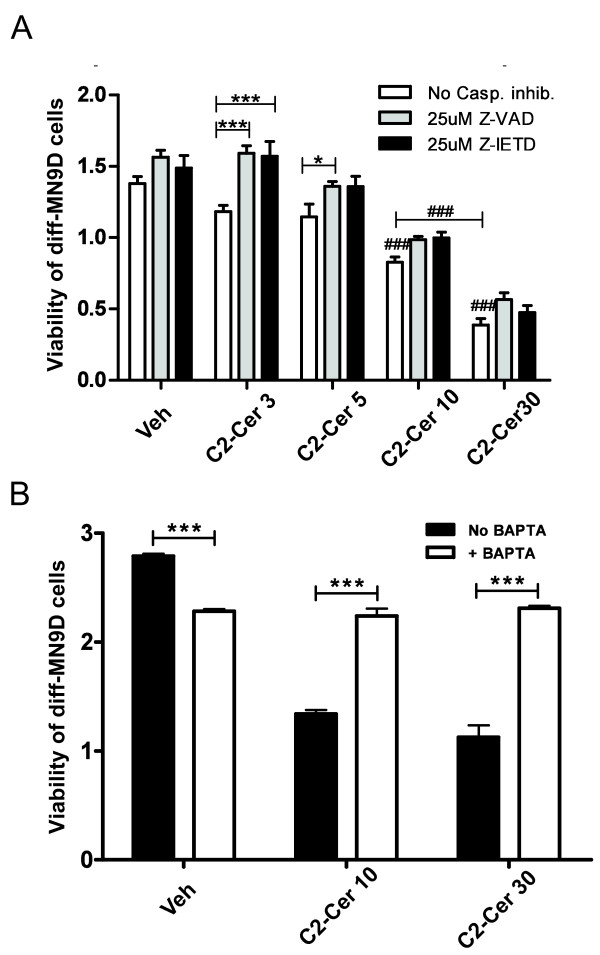
**Disruption of calcium homeostasis (but not caspase signaling) is a primary mechanism by which C2-Ceramide induces cytotoxicity in dopaminergic MN9D cells.A**, The MTS assay for cell viability in diff-MN9D cells. Co-treatment with the pan-caspase inhibitor Z-VAD (25 μM) or the caspase-8-specific inhibitor Z-IETD (25 μM) attenuated cytotoxicity in diff-MN9D cells induced by C2-Cer concentrations at and below 5 μM but not above 5 μM. All values represent group means +/− SEM, n = 3–4. One-way ANOVA with Tukey’s post-hoc test to compare the effect of specified C2-Cer concentrations on diff-MN9D viability to vehicle without caspase inhibitors in the MTS assay, where ### denotes p < 0.001 compared to vehicle or between two C2-Cer concentrations. Two-way ANOVA with Tukey’s post-hoc to compare effects of caspase inhibitors at each C2-Cer concentration where * denotes p < 0.05 and *** denotes p < 0.001 compared to no caspase inhibitor. **B**, Pre-loading of diff-MN9D cells with cell permeant BAPTA-AM (10 μM) to buffer intracellular Ca^2+^ transients significantly attenuated cytotoxicity induced by C2-Cer. Cell viability was assayed by MTS reduction. One-way ANOVA with Tukey’s post-hoc test; *** denotes p < 0.001 compared to no BAPTA at each concentration.

Loss of mitochondrial membrane potential and release of cytochrome C from mitochondria generally precede caspase-dependent apoptotic cell death and a wealth of data has linked TNF bioactivity to caspase activation and apoptosis in various cell types (reviewed in [47]). Similarly, ceramide has been reported to cause apoptotic cell death by altering the Bax/Bcl2 ratio which triggers cytochrome C release from the mitochondria and results in activation of the caspase-9/-3 cascade in C6 glioma cells [48]. Therefore, we investigated the extent to which addition of SMase inhibitors during TNF treatment attenuated caspase signaling. Western blot analyses showed that desipramine and GW4869 significantly attenuated caspase 3 cleavage in TNF-treated diff-MN9D cells (Figure 5A, B). To correlate this finding with TNF-induced cytotoxicity in diff-MN9D cells, we determined the extent to which pan-caspase inhibition (with Z-VAD) or caspase 8 inhibition (with Z-IETD) could ameliorate TNF dose-dependent loss of viability in diff-MN9D. We found that both caspase inhibitors robustly protected diff-MN9D cells from TNF-induced cytotoxicity at all TNF concentrations (Figure 5C), demonstrating that caspase activation is obligate for TNF-induced apoptotic cell death in terminally differentiated MN9D cells and suggesting that TNF-dependent ceramide generation promotes activation of caspase 8 and caspase 3 signaling cascades that lead to apoptotic death in DA cells and neurons. Interestingly, we also found that C2-Cer-induced cytotoxic cell death in diff-MN9D cells was not significantly blocked by Z-VAD or Z-IETD (Figure 6A), which is not entirely surprising since exogenously added C2-Cer would act downstream of TNF/TNFR1-dependent caspase 8 activation. However, we hypothesized that TNF-stimulated ceramide exerts cytotoxicity in DA cells by dysregulating intracellular Ca^2+^ based on reports that implicate defective Ca^2+^ homeostasis in apoptotic cell death of neuronal populations induced by aberrant sphingolipid metabolism [49]. To test this hypothesis directly, we pre-incubated diffMN9D cells with BAPTA-AM prior to exposure to C2-Cer and found that buffering intracellular free calcium nearly ablates C2-Cer-induced toxicity in diff-MN9D cells (Figure 6B), suggesting that elevation of [Ca^2+^_i_ contributes to C2-Cer-induced neurotoxicity.

### *TNF and Ceramide attenuate p-Akt activation to facilitate TNF-induced neurotoxicity in DA cells*

**Figure 7:**
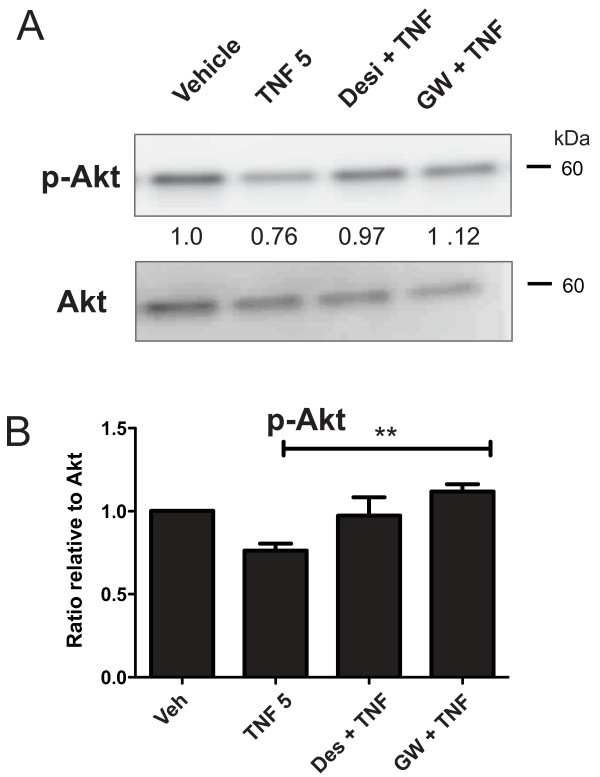
**TNF-induced decreases in phospho-Akt pro-survival signaling were abolished by SMase inhibitors.A**, Diff-MN9D cells were treated with 5 ng/mL TNF in the presence or absence of SMase inhibitors Desipramine (Des 5 μM) or GW4859 (10 μM) and protein lysates were thereafter harvested for SDS-PAGE and immunoblot analysis of phospho-Akt (p-Akt) immunoreactivity. **B**, Quantification of western blot analysis of phospho-Akt (p-Akt) relative to total Akt. One-way ANOVA with Tukey’s post-hoc test, where ** denotes p < 0.01 compared to TNF without inhibitor.

Next, we tested the hypothesis that TNF-dependent ceramide-induced cytotoxicity in diff-MN9D cells may also result from reduced activation of pro-survival pathways, such as Akt signaling. Therefore, we investigated the effect of TNF on phosphorylation of Akt, a key step in pro-survival signaling in the majority of neurons [50,51] We found that TNF treatment reduced p-Akt levels in DA cells and SMase inhibitors robustly blocked this effect (Figure 7). Together with results from caspase inhibition experiments, these data suggest that TNF treatment leads to generation and accumulation of ceramide (and perhaps other downstream sphingolipid metabolites), leading to cytotoxicity in DA neurons via increased ER stress, compromised mitochondrial membrane potential, increased caspase-3 dependent apoptotic signaling cascades, and attenuation of phospho-Akt-dependent pro-survival signaling.

### TNF induces generation of ceramide and atypical sphingoid bases in dopaminergic neuroblastoma cells

**Figure 8:**
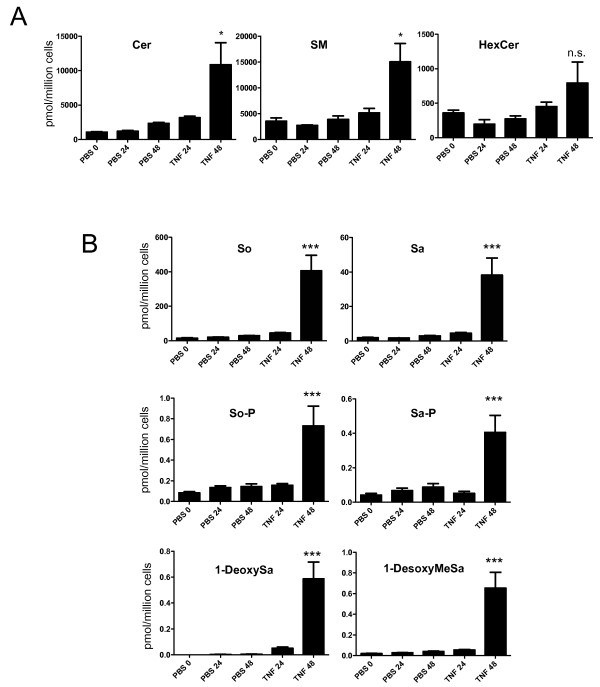
**TNF treatment induces accumulation of ceramide and several atypical sphingoid bases (DBS) in MN9D dopaminergic (DA) neuroblastoma cells derived from mouse ventral mesencephalon.** MN9D cells were treated with PBS or 5 ng/mL TNF for 24 or 48 hours as indicated prior to cell harvest for lipid extraction as described in Methods. **A**, Lipidomic analyses indicate time-dependent accumulation of ceramide (Cer), sphingomyelin (SM), and hexosylceramide (HexCer) in TNF-treated cells relative to PBS-treated cells. **B**, Lipidomic analyses also revealed time-dependent accumulation of sphingosine (So), sphinganine (Sa), sphingosine-phosphate (SoP), sphinganine-phosphate (SaP), 1-deoxysphinganine (1-deoxySa), and 1-desoxymethylsphinganine (1-desoxyMeSa) after treatment with TNF. All values represent group means +/− SEM, n = 3. One-way ANOVA with Tukey’s post-hoc test; *** denotes p < 0.001 and * denotes p < 0.05 compared to PBS at 48 hrs. N.S. denotes not significant.

Given that SMase inhibition affords significant protection from TNF-dependent toxicity in DA neuroblastoma cells and primary DA neurons, it was of interest to confirm that TNF treatment results in detectable formation of ceramide *in vivo.* We used a lipidomics approach to enable quantitative analysis of complex sphingolipids and sphingoid bases in lipid extracts of MN9D cells exposed to PBS or soluble TNF for up to 48 hours. We chose to use DA neuroblastoma cells for our analysis because a homogeneous population of cells is needed for a meaningful result and primary DA neurons only make up a small percentage of total neurons in ventral midbrain cultures. Our analyses indicated that TNF exposure significantly increased the intracellular levels of total ceramide (Cer), sphingomyelin (SM), and hexosylceramide (HexCer) (Figure 8A) as well as several sphingoid bases including sphingosine (So), sphinganine (Sa), sphingosine-1-P (SoP), sphinganine-1-P (SaP), and the atypical sphingoid bases deoxy-sphinganine (deoxySa or DEOSA) and desoxymethylsphinganine (desoxyMeSa or DEOMSA) (Figure 8B). TNF-induced increases in the levels of other complex sphingolipids including deoxydihydro-Ceramide (deoxyDH-Cer) and deoxyceramide (deoxyCer) were not consistently or reproducibly detected (data not shown). These data raise the possibility that in addition to ceramide, any of these additional sphingolipids could be critical second messengers involved in mediating TNF cytotoxicity in DA neuroblastoma cells.

### *Atypical sphingoid bases induce cytotoxicity in differentiated MN9D cells and inhibit neurite outgrowth in primary DA neurons from ventral mesencephalon*

**Figure 9:**
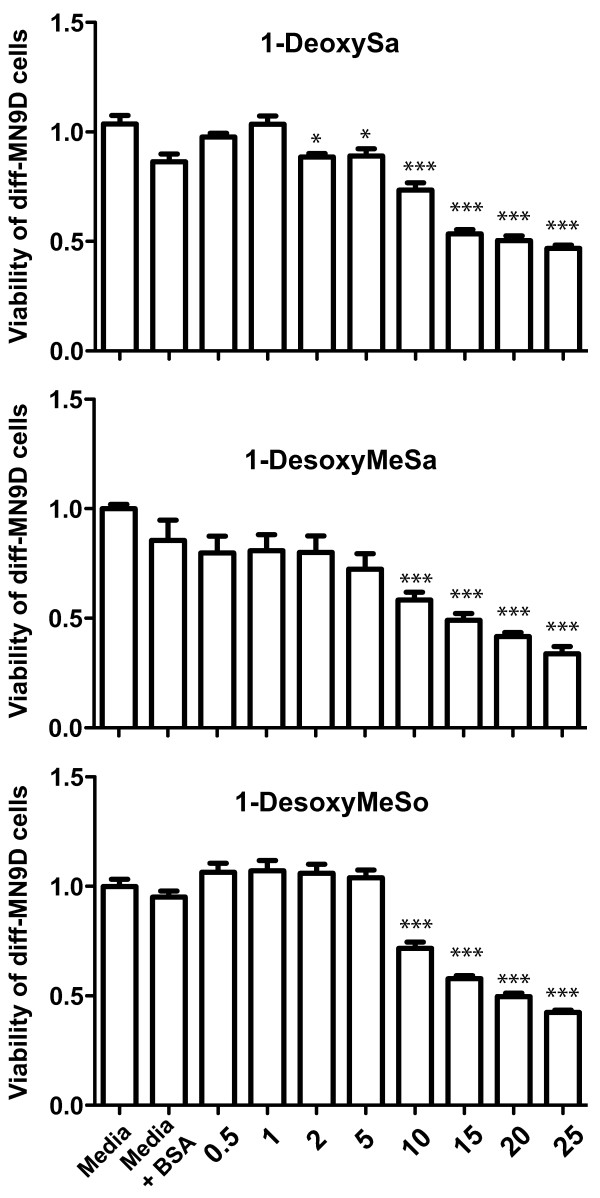
**Atypical sphingoid bases generated in response to TNF exert direct cytotoxic effects on neurally differentiated MN9D dopaminergic (DA) cells.** MN9D cells were plated in 96-well plates, and were neurally differentiated as described under Methods and exposed to treatement media alone (without BSA) or to various doses of 1-deoxysphinganine (1-deoxySa), 1-desoxymethylsphinganine (1-desoxyMeSa), or 1-desoxymethylsphingosine (1-desoxyMeSo) at the concentrations indicated in a complex with BSA (25 μM) for 48 hours. Cell viability was measured by the MTS assay described under Methods. All values represent group means +/− SEM, n = 3–4. One-way ANOVA with Tukey’s post-hoc, * denotes difference from treatment media alone (without BSA) at p < 0.05, *** denotes difference from media alone at p < 0.001.

**Figure 10:**
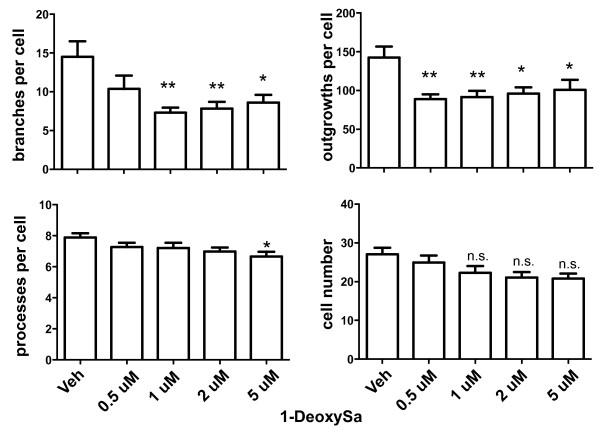
**The atypical sphingoid base 1-deoxySa reduced neuritic branches and outgrowth in primary DA neurons.** Primary neuron-glia cultures from rat ventral mesencephalon were plated in 96-well plates and exposed to treatment media alone without BSA (0) or to 1-deoxysphinganine (1-deoxySa) at the concentrations indicated in a complex with BSA (25 μM) for 48 hours prior to assessing number of branches per cell, number of processes, and number of outgrowths per cell as well as cell number using Image Xpress high-content imaging analyses. 1-DeoxySa was the only one of the sphingoid bases tested that reduced neurite outgrowth and branching. All values represent group means +/− SEM, n = 3–4. One-way ANOVA with Tukey’s post-hoc’ * denotes difference from treatment media alone at p < 0.05, and ** denotes p < 0.01. N.S. denotes not significant.

Based on results from lipidomics analyses (Figure 8B) which indicated that TNF exposure not only increased ceramide levels but also resulted in significant increases in the intracellular levels of several atypical deoxy-sphingoid bases (DSBs), including deoxysphinganine (deoxySa or DEOSA) and desoxymethylsphinganine (desoxyMeSa or DEOMSA), we wanted to test these atypical DSBs for direct cytotoxic effects on cells. These DSBs are devoid of the C1-hydroxyl group of sphinganine and can therefore neither be metabolized to complex sphingolipids nor degraded by the regular sphingolipid catabolism, raising the possibility that they may accumulate within DA neurons and may be cytotoxic. Therefore, we tested the extent to which 1-deoxySa, 1-desoxyMeSa, and 1-desoxyMeSo induce dose-dependent cytotoxicity in diff-MN9D cells and found that all three induced dose-dependent cytotoxicity with an IC50 around 15 μM (Figure 9). To confirm and extend the significance of these findings, we investigated the cytotoxicity of these atypical sphingoid bases on primary cultures from rat ventral mesencephalon. We found that only 1-deoxySa significantly reduced the number of neuritic branches and outgrowths per DA neuron at concentrations as low as 0.5 μM (Figure 10); a trend towards compromising DA neuron viability was also evident but it did not reach statistical significance. No significant cytotoxic effects on primary DA neurons by 1-desoxyMeSa and 1-desoxyMeSo were observed (Additional file 3: Figure S3).

## Discussion

**Figure 11:**
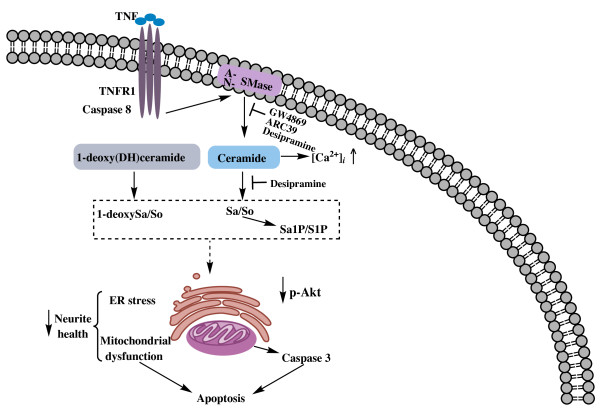
**Proposed model for cellular mechanisms and signaling pathways activated by TNF and ceramide/sphingolipid signaling to induce neurotoxicity in DA neurons.** We propose a model by which TNF/TNFR1-dependent activation of SMases triggers production of ceramide and other downstream lipid metabolites that promote activation of caspase-8/3 signaling, decreased Akt activation and mitochondrial membrane potential, and increased endoplasmic reticulum (ER) stress in DA cells.

The purpose of these studies was to test the hypothesis that ceramide-dependent signaling mediates TNF-induced cytotoxicity and degeneration of DA neurons. Our results indicate that exposure of neurally differentiated DA neuroblastoma cells to soluble TNF induced activation of membrane-bound sphingomyelinases (SMases) and sphingomyelin (SM) turnover resulting in generation of ceramide as measured by lipidomics mass spectrometry. Direct addition of C2-ceramide to DA neuroblastoma cells or primary DA neurons *in vitro* resulted in dose-dependent cytotoxicity, and pharmacological inhibition of SMases with three different inhibitors of SMase function to block ceramide generation during TNF exposure (but not inhibitors of *de novo* ceramide synthesis) afforded significant protection from TNF-induced cytotoxicity. Although desipramine can exert SMase-independent effects on cells [52], two other inhibitors with greater specificity for SMase (GW4869 and ARC39) afforded similar protection against TNF-induced cytotoxicity. Based on these findings, we propose a model by which binding of soluble TNF to TNFR1 on the cell surface of DA neurons activates SMases to generate ceramide and trigger downstream signaling cascades that compromise survival of DA neurons by eliciting ER stress, reducing mitochondria membrane potential, leading to activation of caspase-3-dependent pro-apoptotic signaling and inhibition of Akt-dependent pro-survival signaling cascades which combine to compromise survival of DA neurons (Figure 11). Interestingly, TNF treatment also induced SM biosynthesis (Figure 8A); the significance of this novel finding is unknown, but TNF and lipopolysaccharide (LPS) have both been reported to induce sphingolipid biosynthesis in liver [53] and macrophages [54]. It is also worth noting that increases in atypical deoxy-sphingoid bases (DSBs) were detectable in DA cells after prolonged exposure to TNF (Figure 8B), the potential significance of which is discussed below.

Glycosphingolipid (GSL) metabolism represents a metabolic cross point that interconnects lipid (acyl-CoA) and amino acid (serine and alanine) metabolism. For a detailed review of the metabolic interrelationships that account for the tens of thousands of molecular subspecies in the mammalian sphingolipidome, the reader is referred elsewhere [55,56]. Briefly, ceramide (Cer) consists of a fatty acid acyl chain that varies in length and saturation, and a sphingoid base that differs in the number and position of double bonds and hydroxyl groups. Tissue- and cell type-specific ceramide synthases control the length of the fatty acid chain of ceramide. Sphingoid bases are formed from the precursors L-serine and palmitoyl-CoA in a reaction catalyzed by serine-palmitoyltransferase (SPT). SPT metabolizes other acyl-CoAs besides palmitoyl-CoA but also shows variability towards the use of other amino acid substrates. For instance, SPT is also able to metabolize alanine, which results in the formation of an atypical deoxy-sphingoid base (DSB). These atypical and relatively novel DSBs are devoid of the C1-hydroxyl group of sphingosine (SA) and are therefore neither metabolized to complex sphingolipids nor degraded by the regular sphingolipid catabolism, since sphingosine-1P as a catabolic intermediate cannot be formed from DSBs [34,57]. Missense mutations in SPT long-chain subunit 1 (*SPTLC1*) increase its promiscuous activity towards alanine over serine and result in pathologically elevated DSB levels in the case of the autosomal dominant hereditary sensory and autonomic sensory neuropathy type 1 HSAN1 [34,58]; as evidence of their capacity to induce cytotoxicity, addition of deoxySa to dorsal root ganglion (DRG) neurons in culture can be shown to reduce neurite formation and to disrupt the neuronal cytoskeleton [34]. Given that we observed similar effects in deoxySa-treated DA neurons, we speculate that TNF-stimulated *de novo* synthesis of atypical DSBs may be a secondary mechanism that contributes to TNF-dependent toxicity and reduced viability of DA neurons during inflammatory stress. In fact, neurons may have heightened vulnerability to cellular disturbances in lipid metabolism based on the observation that the majority of GSL lysosomal storage diseases (LSDs) with CNS involvement result in neuronal death, even though the enzymes affected by the gene mutations are expressed ubiquitously [59].

TNF and ceramide have been shown to impinge on endoplasmic reticulum (ER) stress mechanisms in non-neuronal cells types [40,41] and ER stress has been implicated as a potentially important pathway in neurodegenerative diseases [60]; however, whether ER stress is a cause, result, or epiphenomenon in the DA neuron loss that occurs in PD has not been firmly established. ER stress-mediated cell death has been implicated in PD pathogenesis [42,61], being coupled to the cell death program in DA cells in response to the toxin paraquat [43]. Here, we show for the first time that inflammatory signaling through TNF and ceramide induces ER stress in DA neuron-like cells and that SMase inhibition attenuates ER stress and prevents TNF-induced cytotoxicity (as measured independently by MTS and LDH release assays). ATF6 is a direct target of the ER stress response [61] and is known to activate transcription of chaperone proteins [62] to facilitate protein folding and processing capacity; ATF6 also activates ER-associated degradation (ERAD) to promote the degradation of terminally misfolded proteins [63]. Mechanistically, defective calcium homeostasis, especially increased intracellular Ca^2+^ release, presumably from the ER, has been implicated in neuronal cell death in mouse models exhibiting increased CNS glucosylsphingosine levels which can also suppress neuronal outgrowth [49,64,65]. Our data that BAPTA-AM markedly blocked ceramide-induced neurotoxicity is consistent with a role for ceramide as a disruptor of Ca^2+^ homeostasis in DA neurons. Interestingly, a recent study reported that MPTP treatment induced ER stress and decreased AKT phosphorylation via loss of TRPC1-dependent ER Ca^2+^ homeostasis in human dopaminergic neuroblastoma SH-SY5Y cells [66]. Importantly, signs of TNF pathway activation [67,68], ER stress [69,70] and reduced levels of AKT phosphorylation [71] have all been reported in the SNpc of PD patients. Taken together, these findings support the idea that disrupted ER Ca^2+^ homeostasis and compromised Akt pathway activation is a common mechanism by which TNF-dependent inflammation and oxidative neurotoxins compromise survival of DA neurons and lead to development of PD-like features.

Many of the genes associated with PD implicate aberrant mitochondrial function in disease pathogenesis [72] and MPTP and rotenone, which are commonly used in rodents to induce features of parkinsonism, are potent mitochondrial complex I inhibitors [73]. While compromised mitochondrial function has been strongly implicated in PD pathophysiology [72], to date, compromised mitochondrial membrane potential in response to inflammatory stimuli (in this case TNF and C2-Cer) has never been demonstrated in DA cells or DA neurons. Our data demonstrate that TNF and C2-Cer-induced cytotoxicity in diff-MN9D cells correlates closely with reduced mitochondrial membrane potential and treatment with SMase inhibitors reverses these mitochondrial deficits. Similarly, in NGF-differentiated PC12 cells, ceramide signaling has been reported to increase mitochondrial Ca^2+^ levels and to induce ultrastructural alterations [74]. Furthermore, ceramide-induced increases in mitochondrial free calcium were subsequently shown to originate in the ER in a ROS-independent fashion [75]. Our data showing that BAPTA-AM buffering of intracellular free calcium ablates ceramide-induced cytotoxicity in diff-MN9D cells support this kind of model; however, additional studies are needed to determine the source of the cellular Ca^2+^ and/or the extent to which disrupted ER or mitochondrial Ca^2+^ homeostasis plays a causative or synergistic role in TNF or GSL-induced mitochondrial dysfunction in DA neurons [49,64,65].

A role for caspase-dependent apoptotic signaling has been implicated in the death of DA neurons that occurs in PD [76,77] and our findings strongly support a role for caspase 8/caspase 3 signaling as downstream effectors in TNF-dependent death of dopaminergic cells. It should be noted that we observe distinct differences in the overall requirement for caspase signaling in TNF- versus C2-Cer-dependent cytotoxicity in diff-MN9D cells. One reason for this may be that TNF signaling generates ceramide in a physiological range which acts in concert with other TNF receptor-mediated signaling events to trigger downstream caspase-dependent apoptotic processes, whereas addition of exogenous C2-Cer (at concentrations that may not be within a physiological range) artificially bypasses TNF receptor-mediated events and exerts toxic effects by targeting other pathways in addition to mitochondria and caspase inhibition and is not sufficient to attenuate cytotoxicity from this extreme insult. Jurkat T cells require ASMase translocation to plasma membrane lipid microdomains to elicit localized ceramide production and eventual apoptotic cell death [78]. Interestingly, in these cells, ASMase translocation has been shown to occur via two distinct mechanisms: a caspase-dependent mechanism utilized by Fas-L and a previously unrecognized caspase-independent mechanism elicited by short wave ultraviolet irradiation (UV-C). Specifically, it was determined that the caspase-independent mechanism of ASMase translocation led to cell death of Jurkat cells and that UV-C treatment of Jurkat cells activates the sphingomyelin pathway independent of caspase 8 or in the presence of a pan-caspase inhibitor. In this study, the authors note that while ASMase is not a direct target of caspase 8, surface translocation of ASMase activated by Fas-L or other TNF superfamily ligands requires minimal caspase 8 and FADD activation (~2% activation is sufficient). In the case of diff-MN9D cells, exogenous addition of C2-Ceramide bypasses the step of ASMase translocation to lipid microdomains in the plasma membrane as well as the concomitant activation of caspase cascades from the signaling complex assembled in microdomains at the cell membrane that otherwise occurs in response to TNFR1 activation, which is likely to result in toxicity that is caspase-independent. Alternatively, it is possible that exogenous addition of ceramide is sufficient to elicit caspase independent cell death via release of mitochondrial apoptogenic factors, but that engagement of TNFR1 by its ligand TNF leads to SMase-dependent production of ceramide and caspase-dependent cell death of diff-MN9D cells. Lastly, Deerberg and colleagues report that there is a combined requirement of both the ER and mitochondria in the induction of signaling pathways of ceramide-mediated caspase-independent programmed cell death in Jurkat cells [79] and a similar mechanism may be occurring in C2-Ceramide treated diff-MN9D cells. Collectively, our data support a model whereby TNF concentrations in the range that elicit half-maximal cytotoxicity and that correspond to low TNF receptor 1 (TNFR1) occupancy activate SMase to initiate downstream signaling by ceramide and other sphingolipid metabolites, which trigger ER stress, decreased mitochondrial membrane potential, and eventually culminate in the caspase-dependent cytotoxic cell death of DA neurons (Figure 11). Support for this model comes from the multiple studies presented here in which pharmacological inhibition of SMases to block ceramide generation during TNF exposure maintained mitochondrial membrane potential, markedly attenuated TNF-induced ER stress and caspase signaling and restored p-Akt levels in DA cells, thereby promoting significant protection from TNF-induced neurotoxicity.

The histopathophysiological hallmark of Parkinson’s disease (PD) is the formation of intraneuronal aggregation and clustering of α-synuclein and ubiquitinated proteins into inclusions commonly referred to as Lewy bodies (LB) typically found in DA neurons of the substantia nigra pars compacta (SNpc) in the ventral midbrain [80]. Notably, several genes known to be involved in the genetics of Lewy body disease (LBD) or heritable PD share in common the fact that they impinge on ceramide metabolism [81]. Therefore, ceramide metabolism has recently received attention as an emerging pathway involved in LBD [82]. For example, heterozygous loss-of-function mutations of the glucocerebrosidase (GBA) locus have recently been shown to be a potent risk factor for PD [81,83]. GBA catalyzes the dissolution of glucocerebrosidase to ceramide and glucose. The lysosomal storage disease Gaucher’s disease (GD) arises from homozygous mutations in GBA, leading to extreme lysosomal accumulation of GBA substrates and onset of GD symptoms [84]. Interestingly however, GBA substrates do not significantly accumulate in the lysosomes of patients with heterozygous GBA mutations, lending support to the hypothesis that generally disrupted ceramide metabolism, as opposed to specific loss of GBA function, may be an initiating factor in PD [81]. Our data offer a mechanistic link between specific GSL accumulation, ER stress, mitochondrial dysfunction, apoptotic signaling and neuronal death in dopaminergic neurons in response to TNF exposure which may be of significance in PD but perhaps also in other chronic neurodegenerative conditions characterized by elevated levels of TNF and other inflammatory factors. Interestingly, the ASMase inhibitor desipramine induces specific and rapid intracellular degradation of ASMase and concomitant abolishment of enzymatic activity [85]; however, desipramine is a tricyclic antidepressant and its action on neurotransmitters seems to be independent of its effects on ASMase activity. Nevertheless, desipramine has been used in clinical trials to treat depression in PD patients [86]; these trials were very short-lived however, and the effect of desipramine on ceramide signaling was not evaluated as an outcome. Therefore, our data and the data of other groups associating ceramide biology and metabolism with PD warrant future studies examining the potential neuroprotective effects of inhibition of ASMase or NSMase in animal models of PD.

In summary, DA neurons in the substantia nigra pars compacta (SNpc) are preferentially vulnerable to neuroinflammatory stimuli and our group previously demonstrated that chronic inhibition of soluble tumor necrosis factor (TNF) signaling with dominant-negative TNF inhibitors attenuated the loss of nigral DA neurons in models of PD. Our present findings support a molecular pathway by which TNF-dependent ceramide/sphingolipid signaling intermediates compromise survival of DA neurons by inhibiting neurite outgrowth, inducing ER stress, reducing mitochondria membrane potential, activating caspase 3-dependent pro-apoptotic signaling cascades and inhibiting Akt-dependent pro-survival signaling pathways. Additional studies are warranted to explore which specific ceramide metabolites and/or atypical sphingoid bases (or combinations thereof) may represent surrogate biomarkers and/or novel drug targets for development of neuroprotective strategies to halt or delay the progressive loss of DA neurons that lead to the disabling motor fluctuations in patients with PD.

## Competing interests

The authors declare that they have no competing interests.

## Authors’ contributions

TNM designed and performed experiments with MN9D cells to measure cell viability, ER stress, mitochondria membrane potential, and caspase activation; analyzed data and performed statistical analysis; participated in writing and editing of manuscript. XC designed and performed experiments with MN9D cells and primary DA neuron cultures to measure cell viability, ER stress, mitochondria membrane potential, caspase and Akt activation, sphingolipid measurements, and atypical sphingoid base toxicity studies; analyzed data and performed statistical analysis; participated in writing and editing of manuscript. SP performed lipidomics experiments and data analysis; participated in writing and editing of manuscript. AHM participated in preparation of lipid-BSA complexes, directed lipidomics experiments and interpretation of data; participated in writing and editing of manuscript. MGT participated in study design, interpretation of data, writing and editing the manuscript. All authors read, edited, and approved the final manuscript.

## Supplementary Material

Additional file 1 **Figure S1.** Non-diff-MN9D cells are not sensitive to C2-Cer-induced cell death. AlamarBlue assay performed as per manufacturer’s instructions. All values represent group means +/− SEM, n = 3–4. One-way ANOVA, Tukey’s post-hoc test; n.s. denotes no significance. Click here for file

Additional file 2 **Figure S2.** TNF-induced cytotoxicity in diff-MN9D cells does not require de novo biosynthesis of ceramide. Diff-MN9D cells were pre-treated with myriocin (Myr, an inhibitor for serine palmitoyltransferase) or fumonisin B1 (FB1, an inhibitor of ceramide synthase) for 30 minutes prior to addition of TNF for an additional 48 hours. Cell viability was measured via an MTS assay as described under Methods. All values represent group means +/− SEM, n =3 - 4. Two-way ANOVA to test for differences between TNF with inhibitor versus TNF without inhibitor for both Myrocin and Fumonisin B1; there were no significant differences between No MYR and 10 μM MYR at any TNF concentration, as determined by a two-way ANOVA. There was statistically significant TNF-induced death of diff-MN9D cells, as determined by a Tukey’s post-hoc test following a statistically significant one-way ANOVA, where ** denotes p < 0.01, *** p < 0.001 for No MYR conditions relative to vehicle, * denotes p < 0.05 for TNF 10 + No MYR compared to TNF 30 + No MYR, and ### denotes p < 0.001 for 10 μM MYR conditions relative to vehicle. There were no significant differences between No FB1 and 50 μM FB1 at any TNF concentration, as determined by a two-way ANOVA. There was statistically significant TNF-induced death of diff-MN9D cells, as determined by a Tukey's post-hoc test following a statistically significant one-way ANOVA where * denotes p < 0.05, *** denotes p < 0.001 for No FB1 conditions relative to vehicle, ** denotes p < 0.01 for TNF 10 + No FB1 compared to TNF 30 + No FB1, and ### denotes p < 0.001 for 50uM FB1 conditions relative to vehicle, # denotes p < 0.01 for TNF 10 + 50 μM FB1 compared to TNF 30 + 50 μM FB1. Click here for file

Additional file 3 **Figure S3.** The atypical sphingoid bases 1-deoxyMeSa and 1-deoxyMeSo did not exert cytotoxicity on primary DA neurons. Primary neuron-glia cultures from rat ventral mesencephalon were plated in 96-well plates and exposed to treatment media alone without BSA (0) or to 1-desoxymethylsphingosine (1-desoxyMeSo) or 1-desoxymethylsphinganine (1-desoxyMeSa) at the concentrations indicated in a complex with BSA (25 μM) for 48 hours prior to assessing number of branches per cell, number of processes, and number of outgrowths per cell as well as cell number using Image Xpress high-content imaging analyses. All values represent group means +/− SEM, n = 3–4. There were no significant effects from treatment with 1-deoxyMeSa and 1-deoxyMeSo as determined by a one-way ANOVA. Click here for file
